# Physical Growth, Biological Age, and Nutritional Transitions of Adolescents Living at Moderate Altitudes in Peru

**DOI:** 10.3390/ijerph121012082

**Published:** 2015-09-25

**Authors:** Marco Cossio-Bolaños, Rossana Gómez Campos, Cynthia Lee Andruske, Antonio Viveros Flores, Cristian Luarte-Rocha, Pedro R. Olivares, Javier Garcia-Rubio, Miguel de Arruda

**Affiliations:** 1 Department of Physical Activity Sciences, Catholic University of Maule, Av. San Miguel 3605, Talca, Chile; 2 Faculty of Physical Education, State University of Campinas, Avenida Érico Veríssimo, 701, Cidade Universitária Zeferino Vaz, Barão Geraldo, CEP 13.083-851, Campinas, Brazil; 3 Instituto de Actividad Física y Salud, Universidad Autonoma de Chile, 5 Poniente 1670, Talca, Chile; 4 Department of Research, Universidad Científica del Sur, Panamerica Sur Km 19 Villa, Lima, Peru; 5 Group of Interdisciplinary Studies in Health Sciences and Sport, Universidad Autonoma de Chile, Talca 5 Poniente 1670, Chile; 6 Research Network on Human Biological Development, Urb. Amauta C-6, Jose Luis Bustamante y Rivero, Arequipa, Peru; 7 Education Center Antonio José de Sucre, Leon Velarde Street s/n, Yanahuara, Arequipa, Peru; 8 Faculty of Physical Activity, San Sebastian University, General Cruz n 1577, Concepción, Chile

**Keywords:** physical growth, biological age, nutritional transitions, adolescents

## Abstract

*Background*: Peru is experiencing a stage of nutritional transition where the principal characteristics are typical of countries undergoing development. *Objectives*: The objectives of this study were the following: (a) compare physical growth patterns with an international standard; (b) determine biological age; and (c) analyze the double nutritional burden of adolescents living at a moderate altitude in Peru. *Design*: Weight, standing height, and sitting height were measured in 551 adolescents of both sexes (12.0 to 17.9 years old) from an urban area of Arequipa, Peru (2328 m). Physical growth was compared with the international standard of the CDC-2000. Biological age was determined by using a non-invasive transversal technique based on years from age at peak height velocity (APHV). Nutritional state was determined by means of weight for age and height for age. Z scores were calculated using international standards from the CDC-2000. *Results*: Body weight for both sexes was similar to the CDC-2000 international standards. At all ages, the girls’ height (*p* < 0.05) was below the standards. However, the boys’ height (*p* < 0.05) was less at ages, 15, 16, and 17. Biological age showed up in girls at age 12.7 years and for boys at 15.2 years. Stunted growth (8.7% boys and 18.0% girls) and over weight (11.3% boys and 8.8% girls) occurred in both groups. A relationship existed in both sexes between the categories of weight for the age and stunted growth by sex. *Conclusions*: Adolescents living at a moderate altitude exhibited stunted linear growth and biological maturation. Furthermore, adolescents of both sexes showed the presence of the double nutritional burden (stunted growth and excessive weight).

## 1. Introduction

Postnatal growth is a dynamic, complex, and lengthy process that continues throughout infancy, childhood, and adolescence. Each one of these stages is characterized by their own rate of growth. For example, the growth rate is highest during the first year of life. Then, it gradually declines until the onset of the adolescent growth spurt (at about 10 years of age for girls and 12 years for boys) [1].

Generally, adolescence is recognized as a time of great physical, psychosocial, cognitive, and emotional growth. During this stage, anatomical and physiological changes occur. These characterize the onset of puberty. This stage begins with the activation of the hypothalamic-pituitary-gonadal axis and ends with attainment of reproductive capability and acquisition of the adult body composition and habitus [2]. 

Typically, pubertal growth consists of a phase of acceleration, followed by a phase of deceleration, and the eventual cessation of growth with the closure of epiphyses [3].

In this sense, maturation is variable among bodily systems and also in timing and tempo of progress [1]. Thus, the degree of biological maturation of children should be assessed once chronology and intensity of maturation have occurred since these continue throughout puberty. Moreover, they are specific to each adolescent, and they may vary considerably between individuals [4]. This range of variation may be evaluated by means of skeletal, sexual, dental, and somatic maturation. The primary objective of this study was to verify the rate of maturation. Maturation may be interpreted as precocious, normal, or retarded [5]. 

In essence, with regard to the somatic maturation, the literature maintains that with the growth spurt, growth rate increases, reaching a peak (peak height velocity, PHV) at about 12 years in girls and 14 years in boys. Then, it gradually declines and eventually ceases with the attainment of adult stature [4,6]. However, at high altitudes, growth in body size and maturation are delayed and extended into the early twenties when compared to growth of low-altitude populations [7]. In addition, in general, a number of studies have demonstrated that adolescents living in geographical regions at high altitudes show delays in physical growth [8,9,10,11]. In that sense, biological research requires quantitative and qualitative documentation of morphological size, shape, body composition, patterns of growth, and development during infancy, childhood, and adolescence, and rate of change during adulthood [12] 

In fact, research, in general, has been limited to growth and nutritional state of populations living at low and high altitudes. However, little importance has been given to children and adolescents living at moderate altitudes (Arequipa, 2328 m), specifically in countries like Peru. In addition, Arequipa is characterized by a very diverse, rugged territory, with great geographical and altitudinal variations. In addition, it is considered to be the second most developed city after Lima where the Human Development Index [13] for 2013 (0.745) was greater than that of Peru itself (0.741). As a consequence, this accelerated growth brought rapid urbanization to the city. Moreover, this phenomenon may lead to a double nutritional burden in adolescents. This is typical of developing countries where growth patterns and health generally change, especially in terms of going from under weight and retarded growth to overweight and obesity. As a result, this makes Peru attractive and relevant for studying physical growth patterns, biological maturation, and the nutritional state of adolescent students specifically living at a moderate altitude (2328 m).

Thus, the hypotheses of this study are primarily based on observing normal growth and biological maturation, similar to low altitude, and the absence of the double nutritional burden of adolescents studied at moderate altitude. Based on this perspective, to answer the question put forth by the hypothesis, three objectives were proposed: (a) compare the physical growth patterns with an international standard; (b) determine the biological age by using a non-invasive anthropometric technique; and (c) analyze the presence of the nutritional double burden in adolescent students living at moderate altitude in Peru. 

## 2. Methods

### 2.1. Sample

This study is part of a cross-sectional survey. Data were collected through non-probabilistic sampling of adolescent students from three public high schools from the province of Arequipa (Peru). Arequipa is situated at an altitude of 2328 m above sea level, and it is located 1009 km from the capital of Peru (Lima). The climate of the city of Arequipa is predominantly dry during the months of April to November. The weather during the months of December to March is characterized by the presence of clouds and low rainfall. During the course of the year, the relative humidity varies between 46% and a maximum of 70%, and temperatures oscillate between 10 °C and 25 °C.

The Human Development Index of Peru (IDH) for 2013 was 0.741, and in Arequipa, it was 0.745. The province of Arequipa is considered to be the second city of Peru and is an important center of industry, agriculture, and commerce for Peru. According to the projections of the INEI (National Institute of Statistics and Informatics) [14], its number of inhabitants will reach 852.807 (in the city) by 2013. The base of the population pyramid is composed of minors less than 20 years of age. This is 35% of the total population whereas individuals 20–40 years of age make up 34%. Those between 40 and 60 make up 20% of the population, and those older than 60 make up the remaining 11%.

This study was approved by the Ethical Committee of the Instituto del Deporte Universitario (University Sports Institute) at the Universidad Nacional de San Agustín de Arequipa, Peru, under process number CE.005. All parents and guardians agreeing to their children’s participation were given informed consent forms to sign granting their permission for the student assessments. Students without informed consent, those with physical disabilities, and those not attending the day of the anthropometric evaluation (with medical excuses) were excluded from the study.

A total of 551 students of both sexes (312 males and 239 females) were studied. Age ranged from 12.0 to 17.9 years. The sample exhibited a balanced ratio of participants according to age, for example, in males (12 years: 15%; 13 years: 19%; 14 years: 21%; 15 years: 17%; 16 years: 17%; 17 years: 11%) and females (12 years: 20%; 13 years: 17%; 14 years: 18%; 15 years: 25%; 16 years: 11%; 17 years: 9%). Furthermore, socio-economic status of the adolescents was determined according to the type of school students attended. According to Cossio-Bolaños *et al*. [15], in general, Peruvian students attending public schools are for the most part from a middle class socio-economic background. Those attending urban/marginal schools belong to the lower class, and students from rural schools at higher altitudes tend to have a very low socio-economic status. Ethnicity was determined as mixed based on the color of the skin and the presence of a Spanish last name (40% with two indigenous last names, 35% with one last name of Spanish origin, and 15% with two Spanish last names). All anthropometric data were collected during November and December of 2013. The entire process was carried out by three evaluators with extensive experience. Inter-evaluator Technical Margin of Error (ETM) ranged from 1% to 3%.

### 2.2. Measurements

To calculate the age to the decimal, each student’s registered birth age was used. Age was calculated using the date of birth (day, month, year) and the date (day, month, year) the anthropometric measurements were taken. Then, the result was compared to a standard list of ages calculated to the decimal. The administration of each school provided the information.

The “international working group of kineanthropometry” standardized protocol described by Ross and Marfell-Jones [16] was used to measure the anthropometric variables. Weight was measured by using a digital Tanita (Tanita Corporation Japan^®^) scale with a precision of 1.0 kg. Height was measured with a portable estadiometer (Seca GmbH & Co. KG, Hamburg, Germany) with a precision of 0.1 mm in keeping with the Frankfurt horizontal plane. The head-trunk height (Sitting Height) was taken using a wooden bench with a firm back 50 cm in height with a measurement scale of (0 to 150 cm) with a precision to 1 mm. The students’ anthropometric variables were measured without shoes and the least amount possible of clothing (only a light shirt and shorts).

### 2.3. Calculated Variables

Decimal age categories were based on one-year intervals, for example 12.0 to 12.9 years, 13.0 to 13.9 years, 14.0 to 14.9 years, 15.0 to 15.9 years, 16.0 to 16.9 years, and 17.0 to 17.9 years. Reference curves from the CDC-2000 were used to compare physical growth (weight and height) [17]. Prevalence of weight and height for the age were calculated by taking cutoff points of ±2SD as described by Gorstein *et al*. [18]. Delayed growth (stunted) is defined as the height for age minus 2SD below the median reference for the population. Overweight is defined as weight for the age plus 2SD above the median reference for the population. 

A common maturity-assessment technique in longitudinal studies is the determination of years from attainment of peak height velocity (PHV). PHV is an indicator of somatic maturity and reflects the age at maximum growth rate in stature during adolescence (age at PHV, APHV). In the present cross-sectional study, each individual’s years from APHV were predicted using a gender-specific multiple regression equation that included height, sitting height, leg length, chronological age, and their interactions [19]. Leg length was obtained by subtracting sitting height of the height. Thus, a continuous measure of biological age was generated. Biological age groups were constructed using 1-year intervals such that the −1 PHV age groups included observation between −1.49 and 0.50 years from PHV. The equations used for boys included the predictive equation: −29.769 + 0.0003007-Leg Length and Sitting Height interaction −0.01 177. Age and Leg Length interaction +0.01639. Age and Sitting Height interaction + 0.445-Leg by Height ratio, where R = 0.96, R^2^ = 0.915, and SEE = 0.490.

In girls, the predictive equation was Maturity Offset −16.364 + 0.0002309-Leg Length and Sitting Height interaction + 0.006277-Age and Sitting Height interaction + 0.179-Leg by Height ratio + 0.0009428. Age and Weight interaction, where R = 0.95, R^2^ = 0.910, and SEE = 0.499.

### 2.4. Statistical Analysis

The normal distribution of the data was verified by using the Kolmogorov-Smirnov test. Descriptive statistics were used to calculate mean, standard deviation, frequencies, and percentages. The test for independent samples was run to compare both sexes. Z-scores for age and sex were used to compare growth and nutritional status according to the CDC-2000 standards. Chi-squared analysis was used to verify the relationship between nutritional categories (weight for age and height for age). Comparisons between nutritional categories were determined by means of the ANOVA of one variable. The *post facto* test was performed by means of the Bonferroni correction factor. The analyses were conducted with SPSS 16.0 for Windows with a level of significance of 5%.

## 3. Results

Characteristics of the student variables are shown in Table 1 below. Males weighed more, and their standing height and sitting height in relation to females (*p* < 0.05) was greater. On the other hand, males showed a greater delay in biological age when compared with the females.

**Table 1:** Variables of physical growth, body weight and nutritional status of the study sample.

	Males (n = 312)		Females (n = 239)	
Variables	Mean	SD	Mean	SD
Age (years)	14.8	0.09	14.48	0.10
Weight (Kg)	56.2	10.3 *	50.9	7.7
Standing Height (cm)	161.2	8.0 *	153.1	5.5
Sitting Height (cm)	84.4	4.6 *	81.6	3.2
Biological Age (APHV)	15.2	0.8 *	12.7	0.3

Notes: *: *p* < 0.05, biological age was determined by the years from age at peak height velocity (APHV), standard deviation (SD).

**Figure 1:**
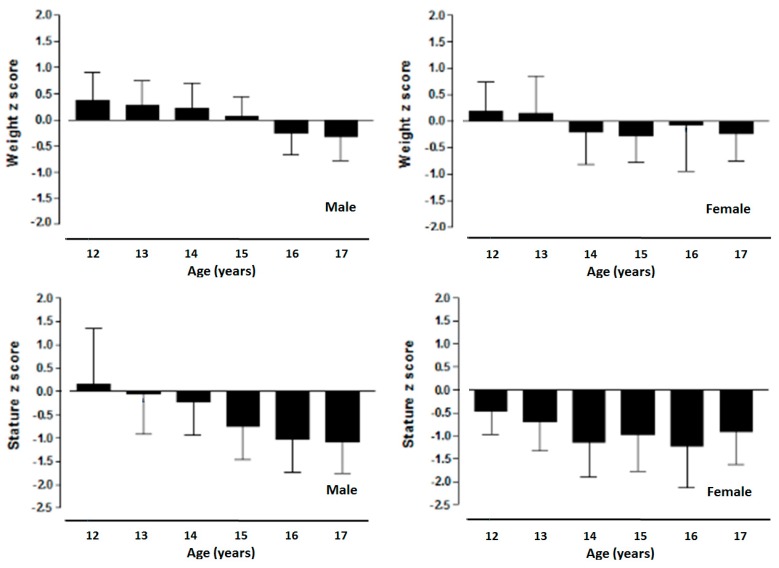
Z-scores for weight and height by age for adolescents of both sexes living in Arequipa (Peru) compared to the CDC-2000 references.

Figure 1 illustrates the Z-scores for weight and height for students living in Arequipa (2328 m). Adolescents of both sexes at all ages showed values relatively similar for body weight when compared to the CDC-2000 international standard. With regard to height, the females of Arequipa (2328 m) showed lower values at all ages in relation to the CDC-2000 standard. On the other hand, males illustrated similar linear growth patterns from age 12 to 14 years, except at ages 15, 16, and 17 where the youth of Arequipa (2328 m) demonstrated lower height in comparison to the international standard. 

**Figure 2:**
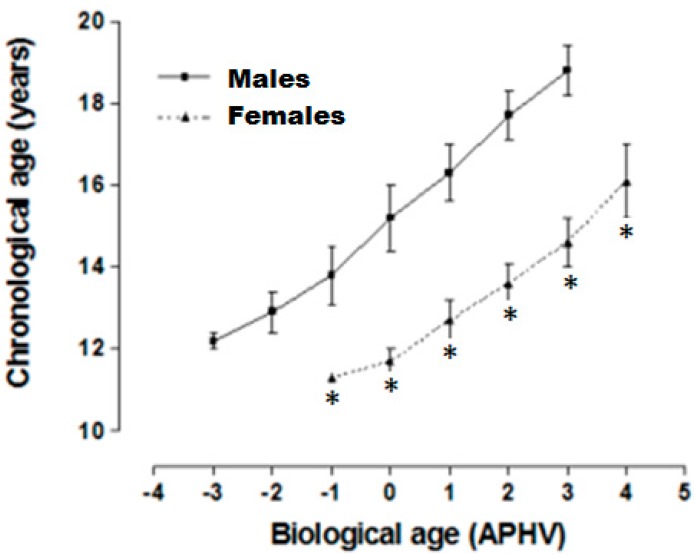
Relationship between chronological age (AC) and biological age (years from age at peak height velocity (APHV) of adolescents of both sexes from Arequipa (2320 m).

Years from age at peak height velocity (APHV) of adolescents of both sexes from Arequipa (2328 m) are shown in Figure 2. Females reached the APHV at 12.7 ± 0.3 years and males at 15.2 ± 0.8 years. Significant differences occurred in both sexes when they were compared by chronological age. Both sexes showed one year of retardation in somatic maturation. 

Table 2 shows the prevalence of nutritional status for the adolescents of both sexes. In the nutritional categories of weight for age, no significant differences were observed (*p* = 0.603). Furthermore, significant differences (*p* = 0.0016) were found in the height categories for age. A greater prevalence of stunted growth was observed in females (18%) than in males (9%).

**Table 2:** Prevalence of nutritional status as a function of weight for age and height for age by sex in adolescents living at a moderate altitude.

Nutritional Status ^1^	Males	Females
**Weight-For-Age**	**Frequency**	**Frequency**
Underweight	13 (4.2%)	11 (4.6%)
Normal Weight	264 (84.5%)	207 (86.6%)
Overweight	35 (11.3%)	21 (8.8%)
**Height-For-Age**		
Stunted	27 (8.7%)	43 (18.0%)
Non-Stunted	285 (91.3%)	195 (82.0%)

Notes: Chi-square test for Weight for age 0.909, 2df (degrees of freedom), *p* = 0.60347; Chi-square test for Height for age 9.94, 1df (degrees of freedom) ( *p* = 0.0016. ^1^ = Z-scores (CDC-2000).

The association between retarded linear growth and nutritional categories for weight based on age are shown in Table 3. In males, females, and both sexes, a relationship occurred between both variables. In addition, females with stunted growth showed a greater tendency for being overweight (2.9%) than did the males (1.6%).

**Table 3:** Association of stunted growth with nutritional categories by weight for age of adolescents of both sexes.

	Weight-For-Age				
Stunted	Underweight	Normal	Overweight	Total	*p*-Value
Males (n = 312)					
Stunted	6 (1.9%)	16 (5.1%)	5 (1.6%)	27 (8.6%)	0.0001
Non-Stunted	7 (2.2)	248 (79.5%)	30 (9.6%)	285 (92.2%)	
Females (n = 239)					
Stunted	5 (2.1%)	31 (13.0%)	7 (2.9%)	43 (18.0%)	0.0043
Non-Stunted	6 (2.5%)	177 (74.0%)	13 (5.4%)	196 (82.0%)	
*Both*-*Sex* (n = 551)					
Stunted	11 (1.9%)	31 (8.5%)	7 (2.2%)	43 (12.6%)	0.0001
Non-Stunted	13 (2.4%)	177 (77.1%)	13 (7.8%)	196 (90.3%)	

Notes: Chi-square test for males 26.80, 2df; for females 10.90, df, and both sexes 31.08, 2df.

Table 4 shows the comparison between both nutritional groups based on stunted growth. Significant differences were found between the three nutritional categories and both Stunted and Non-Stunted in both sexes. In general, overweight subjects identified as Stunted, and Non-Stunted subjects showed higher weight values in relation to other nutritional categories. 

**Table 4:** Comparison between stunted growth and nutritional groups by weight for age.

	Weight-For-Age									
	Underweight			Normal			Overweight			
	N	X	SD	N	X	SD	N	X	SD	*p*-Value
Males										
Stunted	6	44.8	7.79	16	50.84	8.92	5	80.46	1.61	0.0001
Non-Stunted	7	45.3	6.07	248	54.80	8.01	30	70.15	11.64	0.0001
Females										
Stunted	5	42	3.03	31	50.89	6.32	7	77.1	2.25	0.0001
Non-Stunted	6	40.57	2.76	177	50.96	6.30	13	65.67	5.07	0.0001
*Both*-*Sexes*										
Stunted	11	43.40	5.41	47	50.87	7.62	12	78.78	1.93	0.0001
Non-Stunted	13	42.93	4.42	425	52.88	7.16	13	67.91	8.36	0.0001

## 4. Discussion

### 4.1. Physical Growth

The results indicate that the adolescents of both sexes from Arequipa demonstrated similar body weight in relation to the international standards of the CDC-2000. Furthermore, girls experienced stunting in linear growth from ages 12 to 17. In boys, this phenomenon showed up beginning at age 15 until age 17. In essence, the results indicate linear growth stunting occurred during adolescence for students living at a moderate altitude. 

These findings are not unusual as they are supported by the literature given that, after Guatemala, Bolivia, and Haiti, Peru is a country that is characterized by reports of the high prevalence of retarded infant growth [20]. Moreover, it is one of the countries with the highest ratios of iron-deficiency, anemia, and vitamin “A” deficiency in South America [21]. 

In this sense, despite the current reports of the presence of retarded physical growth in Peruvian children, it is necessary to highlight that recently Urke *et al*. [22] maintained that during the last 20 years, a significant statistical decrease in stunted infant growth has occurred. In fact, a few years ago, extensive economic development and improved health care took place in Peru [23]. During the decades of the1980s, 1990s, and 2000, social programs were introduced into state schools to improve the nutritional state of children and adolescents. However, these were not sufficient enough to improve the genetic potential of the adolescents in this study. Based on the results of our research, during this important stage of these adolescents’ lives, the students of Arequipa were affected by delays in their linear growth. However, it should be noted that a recent study [24] of adolescents of both sexes living in Peru at a low altitude verified these results with similar linear growth patterns when compared to the international standard of the CDC-2000. These results suggest normal growth for weight for age and height for age. Therefore, these findings at a low attitude make us assume that these adolescents in this current study (moderate altitude) experience delayed linear growth. This phenomenon can also be seen in boys at an advanced age and girls at all ages. Therefore, this occurs throughout the entire stage of adolescence. 

The patterns observed in this study may be associated with genetic factors specific to this particular group of students with height less than the international standard. However, this phenomenon may be related to environmental factors such as altitude even though the literature supports the view that an altitude greater than 2500 is where the majority of people show an important drop in oxygen saturation [25] that could considerably affect linear growth patterns.

### 4.2. Biological Age

With regards to biological age, what stands out in both sexes is evidence of delayed somatic maturation based on the APHV of the girls at 12.7 ± 0.3 years and at 15.2 ± 0.8 years for boys. In general, the timing and magnitude of the PVC are very variable between adolescents. For example, it is known that the PVC for total height during puberty for 95% of girls tends to begin between the ages of 10–14 and between ages 12 to 14 for 95% of boys [4,26]. However, during this process, somatic growth like maturation is influenced by various factors that act independently or in combination to modify the individual’s genetic potential [27].

In essence, in light of the findings we obtained, the adolescents living in Arequipa at moderate altitude reached APHV one year after the general population. This delay is basically related to the low height observed in this study since adolescents of both sexes were not able to reach their maximum genetic potential. Thus, this delay in physical growth and somatic maturation may be determined by environmental factors like altitude even though other possible intervening environmental factors cannot be discounted as indicated above. Furthermore, it should be noted that other studies related to biological maturation of Peruvian adolescents living at low, moderate, and high altitudes could not be found. These would have been relevant and helped us provide a better explanation of our results. In fact, the patterns of growth and biological maturation in the adolescents studied here were expected to be normal. However, the results obtained indicated the opposite. Based on the findings of this study, what this means is that more in-depth research is needed on these biological variables.

This means that more studies are needed in order to confirm these findings. Moreover, in addition to evaluating anthropometric characteristics, it is necessary to include other physiological and biological variables to verify more precisely in order to explain the delay in physical growth and biological maturation of Peruvian children and adolescents living at diverse altitudes.

### 4.3. Nutritional Transition

The delay in height observed in this study is represented by 8.7% of boys and 18% of the girls. Evidently, 91.3% of the boys and 82% of the girls showed normal linear growth. However, one factor identified that stands out is the presence of elevated weight for age in the adolescents of both sexes. For example, this was observed in 11.3% of the boys and 8.8% of the girls. Therefore, these results clearly reflect a nutritional double burden in the adolescents studied here.

This so-called double burden of malnutrition is typical of countries with low and medium incomes [28,29]. Peru is no stranger to this situation since overweight and stunting in growth constitute a polarized and prolonged transition model [30]. As a result, the Peruvian population is at high risk for developing illnesses associated with both nutritional extremes [20].

In this sense, our study points to the predisposition of these adolescents to the double nutritional burden since there was a relationship between the categories of height and body weight (Table 3) as illustrated by subjects with low height and at the same time elevated body weight for age for both sexes (16% in males and 3% in females). In addition, the adolescents of both sexes with stunting reflected elevated body weight in relation to other nutritional categories. These results are consistent with other studies that highlight the association between short stature and weight in children [31,32]. Furthermore, recent reports suggest that children with delayed growth tend to show alterations in body composition and the distribution of body fat [33,34]. These changes may be related to lower rates of fat oxidation that children with growth retardation tend to have [35] in this way contributing to the increase in the levels of body fat during the course of life [36].

In this study, it was not possible to evaluate other anthropometric variables like skin folds and abdominal perimeter. These would have been important for explaining the predisposition for excessive body fat in adolescents with short stature in this study. Additionally, Body Mass Index (BMI) was not used because some studies maintain that the BMI is not applicable to populations characterized by a predisposition to low stature [15,37]. Therefore, their applicability with this type of population is very questionable, and it could bring the consequence of overestimation of excessive body weight, not only in children and adolescents but also adults. 

Therefore, the cause of the double nutritional burden observed in this study may be explained by the rapid economic growth occurring in Peru [22]. Above all, since in the last 20 years, Peru has rapidly been converted into a country with a high average income [38] in relation to its neighboring countries.

This study has some limitations. For example, no information was included about the physical activity of the adolescents studied. Furthermore, the use of the CDC-2000 reference to study growth as well as nutritional status may show some type of systematic bias in the cutoff values used. Biological age was determined by a non-invasive technique that measures years of PHV transversally. This method was used due to its feasibility in evaluating sexual maturity non-invasively because serious problems could have arisen due to disgust and shame in adolescents, as well as their parents, because of the closed cultural environment of populations living in the highland regions of Peru. In addition, evaluating the oxygen saturation levels is important, especially when studying physical growth and biological maturation at moderate to high altitudes. We suggest using this as a control for future research studies. 

However, despite the limitations indicated above, this study provides relevant data about growth patterns, biological maturation, and nutritional status of adolescent Peruvians. Furthermore, this study indicates future areas for continuing research about cities at moderate altitudes whose primary characteristics have to do with the increased social, economic, and urban development. This would allow for a greater in-depth analysis of not only the effects of altitude but also of the boundaries of nutritional transition. 

## 5. Conclusions

These results suggest the presence of delay in linear growth at more advanced ages in boys and throughout all of adolescence in girls. Moreover, somatic maturation was delayed by one year in both sexes. The double nutritional burden found (retarded growth and excessive weight) in adolescents living at moderate altitude suggests that constant observation is needed in both the positive and negative tendencies in their nutritional state. 
